# Antifungal use and appropriateness: a study of Australian Hospital National Antimicrobial Prescribing Survey data

**DOI:** 10.1017/ash.2025.10214

**Published:** 2025-11-17

**Authors:** Anna Khanina, Nikhil Singh, Josephine Wen, Caroline Chen, Rodney James, David CM. Kong, Monica A. Slavin, Karin A. Thursky

**Affiliations:** 1 The National Centre for Infections in Cancer, Peter MacCallum Cancer Centre, Melbourne, VIC, Australia; 2 Department of Infectious Diseases, Peter MacCallum Cancer Centre, Melbourne, VIC, Australia; 3 Sir Peter MacCallum Department of Oncology, The University of Melbourne, Melbourne, VIC, Australia; 4 The National Centre for Antimicrobial Stewardship, Department of Infectious Diseases, University of Melbourne, The Peter Doherty Institute for Infection and Immunity, Melbourne, VIC, Australia; 5 The Royal Melbourne Hospital Guidance Group, Royal Melbourne Hospital at the Peter Doherty Institute for Infection and Immunity, Melbourne, VIC, Australia; 6 Centre for Medicine Use and Safety, Monash Institute of Pharmaceutical Sciences, Monash University, Parkville, VIC, Australia

## Abstract

**Objectives:**

To evaluate the quality of systemic antifungal prescribing in Australian hospitals using point-prevalence survey data from the Hospital National Antimicrobial Prescribing Survey (Hospital NAPS).

**Methods:**

Data were extracted from the Hospital NAPS dataset from January 2014 to December 2024. Systemic antifungal prescriptions were analyzed for antifungal use, guideline compliance, appropriateness, and reasons for inappropriateness according to the Hospital NAPS methodology. Demographic factors, hospital classifications, antifungals, and antifungal indication were compared.

**Results:**

A total of 7,830 systemic antifungal prescriptions from 372 healthcare facilities across all Australian states and territories were included. Overall 88.4% were guideline compliant and 92.3% of prescriptions were deemed appropriate. Fluconazole was the most commonly prescribed antifungal but had one of the lowest percentage appropriateness (88.4%). In contrast, mold-active azoles, echinocandins, and amphotericin B demonstrated appropriateness rates exceeding 90%. Prescriptions with approval through local antimicrobial stewardship (AMS) processes had significantly higher appropriateness than those without (95.9% vs. 82.9%, *p* < 0.001). Specialized facilities managing immunocompromised populations showed both higher antifungal use and higher prescribing quality compared to general acute public hospitals.

**Conclusion:**

This national evaluation highlights the overall high quality of systemic antifungal prescribing in Australian hospitals, reflecting the strength of AMS programs. However, variation across hospital types, specialties, and antifungal agents—particularly fluconazole—indicates opportunities for targeted stewardship interventions to further optimize antifungal use.

## Background

Invasive fungal infections (IFI) predominantly affect immunocompromised and critically ill patients, contributing to significant morbidity, mortality,^1–5^ and healthcare costs.^6,7^ Studies evaluating antifungal prescribing have reported that a substantial proportion of prescriptions are inappropriate.^8–13^ Inappropriate or unnecessary antifungal use increases the risk of treatment failure, adverse drug reactions, and clinically significant drug–drug interactions, all of which can result in patient harm. Furthermore, the emergence of resistant fungal pathogens, coupled with a growing population of immunosuppressed individuals,^14^ highlights the need for quality improvement initiatives to optimize antifungal prescribing, improve IFI management and patient outcomes.

In Australia, the National Antimicrobial Prescribing Survey (NAPS) is a widely adopted online auditing program that enables hospitals, healthcare centers, and aged care homes to evaluate antimicrobial prescribing practices.^15^ It is a major component of Australia’s national antimicrobial resistance strategy and contributes data to the Antimicrobial Use and Resistance in Australia surveillance program.^16^ The NAPS program plays a key role in assisting healthcare institutions to meet antimicrobial stewardship (AMS) accreditation requirements.^17^ Since its inception in 2013, the Hospital NAPS, a hospital-focused point-prevalence survey (PPS), has supported facilities in monitoring antimicrobial use, assessing guideline compliance, and evaluating prescribing appropriateness.

Antifungal stewardship (AFS) encompasses interventions designed to promote the appropriate use of antifungals, with the goal of enhancing clinical outcomes while minimizing toxicity, resistance, and unnecessary healthcare expenditure. Both national ^18^ and international guidelines ^19^ emphasize the importance of assessing antifungal prescribing quality and implementing data-driven strategies to support continuous improvement.

While the Hospital NAPS data set has been utilized to assess antimicrobial prescribing quality across various therapeutic areas,^20–25^ a comprehensive analysis specifically focused on systemic antifungal prescribing has not yet been conducted. The Hospital NAPS provides a comprehensive national data set that enables detailed interrogation of antifungal prescribing practices across Australian healthcare settings, offering valuable insights to inform AFS strategies and policy development.

The aim of this study was to utilize the Hospital NAPS data set to assess the quality of systemic antifungal prescribing within Australian hospitals over an eleven-year period. Specifically, we sought to compare prescribing practices across different healthcare settings, clinical specialties, and antifungals, and to identify targets for quality improvement. Additionally, we evaluated the strengths and limitations of the PPS methodology for assessing antifungal prescribing quality and its role in supporting AFS efforts.

## Methods

Deidentified data were extracted from the Hospital NAPS database for January 1, 2014, to December 31, 2024. Local hospital auditors submitted deidentified patient information via a secure online platform. Audit methodologies included; whole facility PPS, half-facility PPS in which alternate patients were audited (large hospitals with limited resources) and repeat PPS (smaller hospitals to attain the minimum 30 prescriptions required). The survey excludes outpatients, day procedure patients, and non-admitted emergency department presentations. Participation in Hospital NAPS is voluntary. Ethics approval for this study was granted by the Melbourne Health Human Research Ethics Committee (HREC/74195/MH-2022).

**Figure 1.**
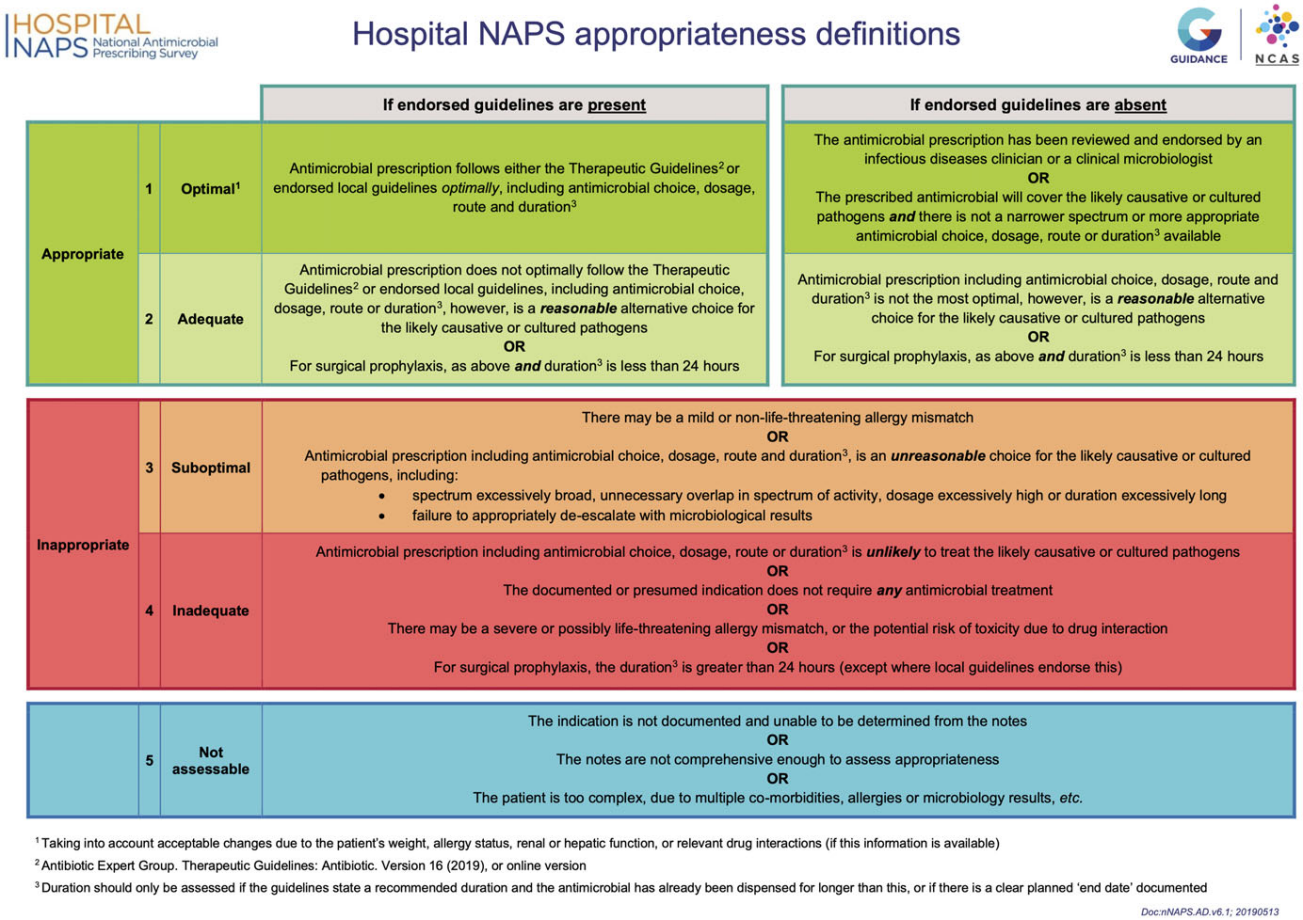
Hospital NAPS appropriateness definitions. Table outlining criteria for appropriateness assessment.

The data collection tool can be viewed in Appendix 1. Each prescription was assessed for guideline compliance, which was evaluated against the Australian national guidelines ^26^ or locally endorsed guidelines, defined as guidelines approved by a local drug and therapeutics committee. Additionally, prescriptions were assessed for appropriateness and, if applicable, reasons for inappropriateness, using a structured matrix (Figure 1). Prescriptions were categorized as optimal, adequate, suboptimal, inadequate, or not assessable.^15^ Assessments were performed at participating facilities by AMS personnel, including infectious diseases physicians, pharmacists, clinical microbiologists, medical practitioners, nurses, or infection prevention and control practitioners, all of whom had completed an eLearning module. A user guide and case examples were available to support consistent assessments. Additional support was provided via email and phone.

Guideline compliance analysis excluded prescriptions classified as “directed therapy,” “no guideline available,” or “not assessable,” thereby focusing the analysis on prescriptions where guideline compliance could be assessed. The NAPS user guide instructs users to select “directed therapy” where an antimicrobial is directed according to microbiology culture or susceptibility results, with users not able to assess guideline compliance further. Appropriateness classifications were grouped into three categories: appropriate (optimal, adequate), inappropriate (suboptimal, inadequate), and not assessable. To ensure accurate representation of evaluable prescriptions, appropriateness rates were calculated after excluding those marked as “not assessable.” Analysis of reasons for inappropriateness were restricted to prescriptions deemed inappropriate.

For this analysis, only systemic antifungals, defined as those administered orally or intravenously and approved for use in Australia, were included. Oral nystatin was excluded due to its low bioavailability. Antifungals were grouped by spectrum of activity into the following categories: mold-active azoles (isavuconazole, itraconazole, posaconazole, voriconazole), echinocandins (anidulafungin, caspofungin, micafungin), and amphotericin B formulations (conventional, lipid complex, liposomal). All other antifungals were analyzed individually.

Participating hospitals were classified as public or private. Public hospitals are government-funded and provide care at minimal or no cost to patients, while private hospitals are independently operated, with costs covered by private health insurance, government subsidies, and patient contributions. Hospitals were further categorized by remoteness, distinguishing between major cities and regional or remote areas, using the Australian Statistical Geographical Standard.^27^ Hospitals were also categorized by the Australian Institute of Health and Welfare peer group classification, according to their specialty services and patient casemix.^28^ Of particular note is the principal referral classification which is assigned to large public acute hospitals that provide a very broad range of services, including specialized units and have large patient volumes. Treating specialties were grouped into hematology and bone marrow transplant, oncology (medical, surgical, and radiation), medical, surgical, and solid organ transplant. Records associated with critical care or pediatric specialty were excluded from specialty-based comparisons due to inconsistent documentation of these two specialties in the data set.

Statistical analyses were conducted using Stata version 16.1 (StataCorp, College Station, TX). Descriptive statistics were used to summarize data, and comparisons were made using the chi-squared test, with a *P* value of <0.05 considered statistically significant.

## Results

A total of 324,784 antimicrobial prescriptions were submitted from 649 healthcare facilities across Australia between 2013 and 2024. Of these, 7,830 prescriptions were for systemic antifungals, originating from 372 facilities. All Australian states and territories were represented in the data set. Patient, facility, and prescribing specialty characteristics are summarized in Table 1.

**Table 1.** Antifungal prescribing distribution

	n (%)
**Demographics**	
Age, median y (IQR)	61 (43–71)
Sex, male	4462 (56.99)
**Peer group**	
Principal referral hospitals	4156 (53.00)
Acute public hospitals	1633 (20.86)
Acute private hospitals	904 (11.55)
Women’s and children’s hospitals	816 (10.42)
Other specialized acute hospitals	167 (2.13)
Subacute hospitals	124 (1.58)
Other hospitals	30 (0.38)
**Remoteness**	
Major cities	6794 (86.77)
Regional and remote	1036 (13.23)
Specialty group*	
Hematology and bone marrow transplant	2972 (39.41)
Medical	2188 (29.01)
Surgical	1351 (17.92)
Oncology	799 (10.60)
Solid organ transplant	231 (3.06)

* Intensive care unit and pediatric specialties excluded from analysis.

### Antifungal use

Over the eleven-year surveillance period, systemic antifungal prescriptions accounted for 2.41% of all antimicrobial prescriptions. More than half of all systemic antifungal prescriptions were contributed by principal referral hospitals, followed by acute public hospitals and acute private hospitals (Table 1). A greater proportion of antifungal use was observed in major cities compared to regional and remote areas. Hematology and bone marrow transplant units accounted for the highest proportion of systemic antifungal use.

### Guideline compliance and appropriateness

**Figure 2.**
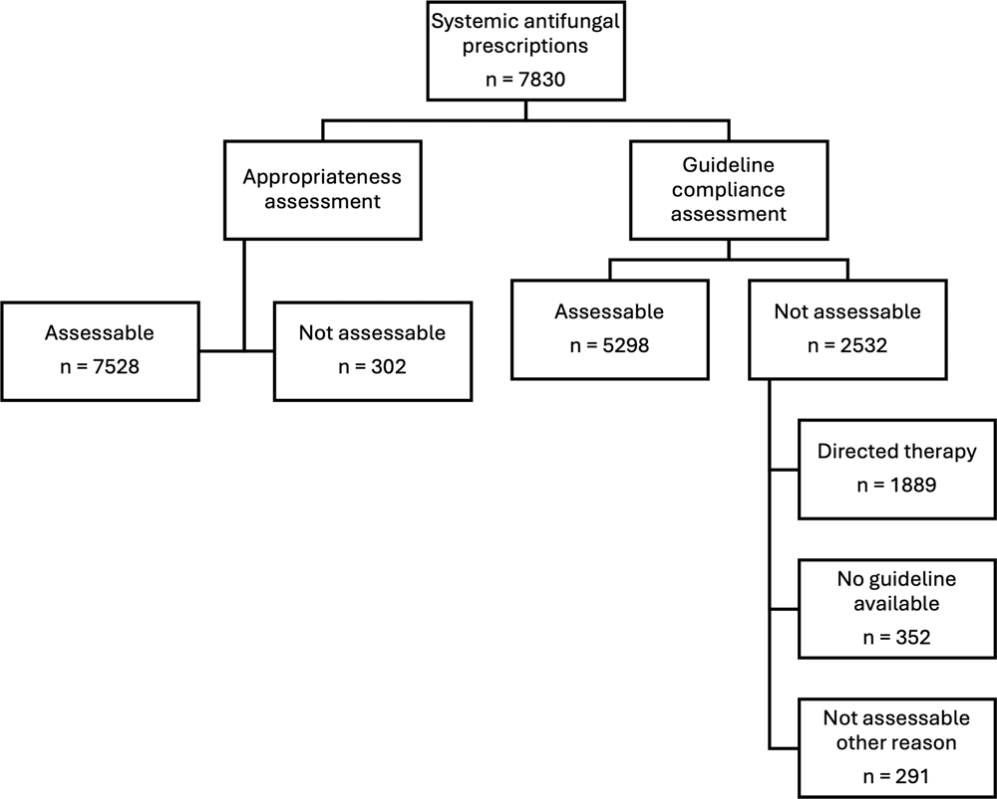
Prescriptions excluded from appropriateness and guideline compliance analysis. Flowchart showing the assessment process for 7,830 systemic antifungal prescriptions.

Of the 7,830 systemic antifungal prescriptions, 32.34% were not assessed for guideline compliance due to being classified as directed therapy, having no applicable guideline, or being otherwise not assessable (Figure 2). Among the prescriptions where guideline compliance could be assessed, 88.39% were compliant. Higher rates of guideline compliance were observed in specialized centers, including principal referral, women’s and children’s, and other specialized hospitals. Lower compliance rates were noted in regional and remote centers. Specialty units managing immunocompromised patients demonstrated higher rates of compliance (Table 2).

**Table 2.** Appropriateness and guideline compliance where assessed

	Guideline compliance n (%)	Appropriateness n (%)
Number of assessable prescriptions	5 298	7 528
Overall	4683 (88.39)	6950 (92.32)
**Peer group**		
Principal referral hospital	2618 (92.51)	3820 (94.18)
Acute public hospital	750 (78.45)	1334 (87.19)
Acute private hospital	502 (80.19)	754 (91.28)
Women’s and children’s hospital	581 (93.11)	758 (91.28)
Other specialized acute hospital	150 (96.77)	160 (96.39)
Subacute hospital	65 (77.38)	103 (89.57)
Other hospital	17 (73.19)	21 (87.50)
**Remoteness**		
Major cities	4177 (90.01)	6123 (93.22)
Regional and remote	506 (76.67)	827 (86.51)
Specialty group*		
Hematology and bone marrow transplant	2590 (95.78)	2841 (96.63)
Medical	974 (78.17)	1781 (87.91)
Surgical	300 (71.26)	1154 (89.67)
Oncology	542 (88.27)	718 (92.41)
Solid organ transplant	136 (88.89)	198 (89.59)

* Intensive care unit and pediatric specialties excluded from analysis.

**Figure 3.**
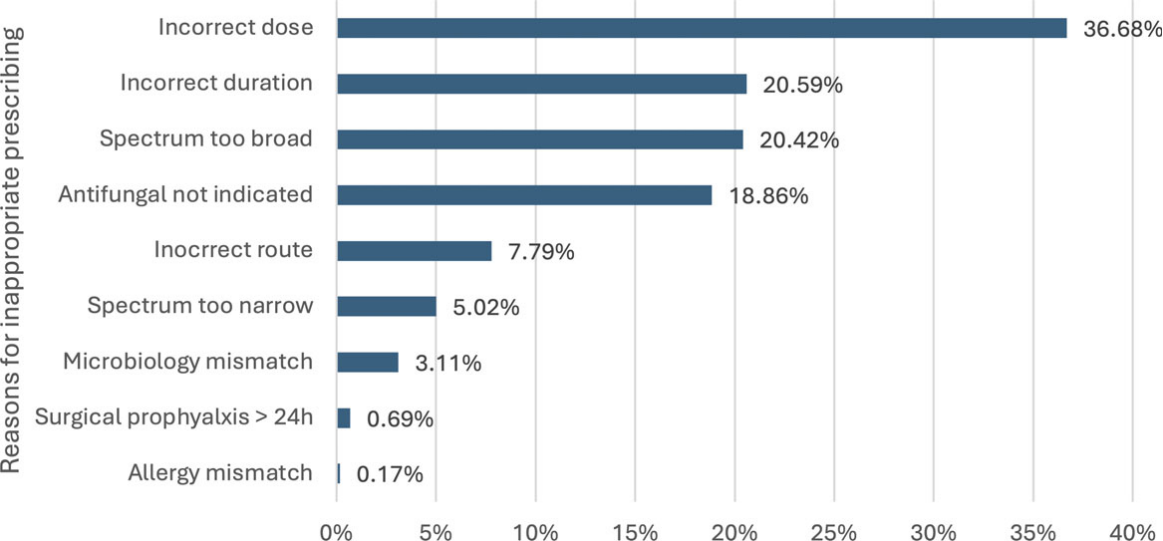
Reasons for inappropriate antimicrobial prescribing. Bar chart showing the proportion of prescribing errors categorised by type.

For appropriateness, 3.86% of prescriptions were deemed not assessable (Figure 2). Among assessable prescriptions, 92.32% were considered appropriate. Appropriateness rates were lower in acute public compared to principal referral hospitals. Appropriateness was higher in major city compared to regional and remote centers. The highest rates of appropriate prescribing were observed in hematology and bone marrow transplant units (Table 2). Among prescriptions assessed as inappropriate, the most common reasons were incorrect dose, incorrect duration, or overly broad spectrum of activity (Figure 3).

### Appropriateness by antifungal group

**Figure 4.**
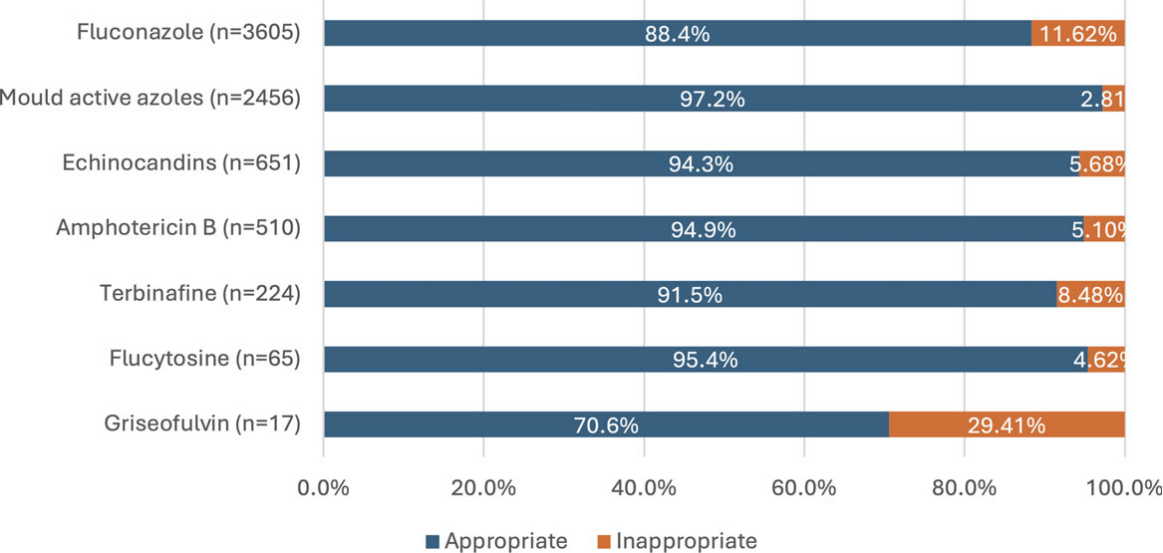
Appropriateness of antifungal prescribing by agent/class. Bar chart showing the proportion of appropriate and inappropriate use for each antifungal agent.

Fluconazole was the most frequently prescribed antifungal, accounting for 48.35% of all prescriptions. It had one of the lowest percentage appropriateness (Figure 4). The most common indication was medical prophylaxis, with a high appropriateness of 94.51%. Fluconazole prophylaxis was predominantly prescribed in hematology, oncology, or solid organ transplant settings where appropriateness was above 90% for each speciality. Use by medical specialities represented 10.2% of fluconazole prophylaxis prescriptions with an appropriateness of 85.2%. The second most common indication was urinary tract infections, with 85.29% of prescriptions assessed as appropriate. Common reasons for inappropriateness in urinary tract infections included incorrect dosing and antifungal therapy not indicated. Oral and dental infections were the third most frequent indication, with the lowest appropriateness (72.73%), primarily due to incorrect dose and duration.

Mold-active azoles were the second most prescribed antifungal group and had the highest overall appropriateness (Figure 4). Medical prophylaxis was the most common indication (97.59% appropriate), followed by respiratory infections (95.25% appropriate). Inappropriate use was most often attributed to incorrect dosing, antifungal not indicated, or overly broad spectrum of activity.

Echinocandins accounted for 8.45% of all prescriptions and had high appropriateness. The most common indications were sepsis and fungemia, medical prophylaxis, and intra-abdominal infections, all with appropriateness exceeding 90.00%. The most common reason for inappropriateness was overly broad spectrum of activity, followed by incorrect duration and antifungal not indicated.

Amphotericin B formulations represented 6.60% of prescriptions and had a high appropriateness. The most common indications were medical prophylaxis, systemic infections, and central nervous system infections. Inappropriate use was most often due to overly broad spectrum of activity, incorrect dosing, or incorrect duration.

Terbinafine accounted for 3.10% of prescriptions. The primary indication was skin and soft tissue infections. Inappropriate use was mainly due to incorrect duration and dosing. Flucytosine and griseofulvin were prescribed infrequently and were therefore excluded from detailed analysis.

### Process measures

**Figure 5.**
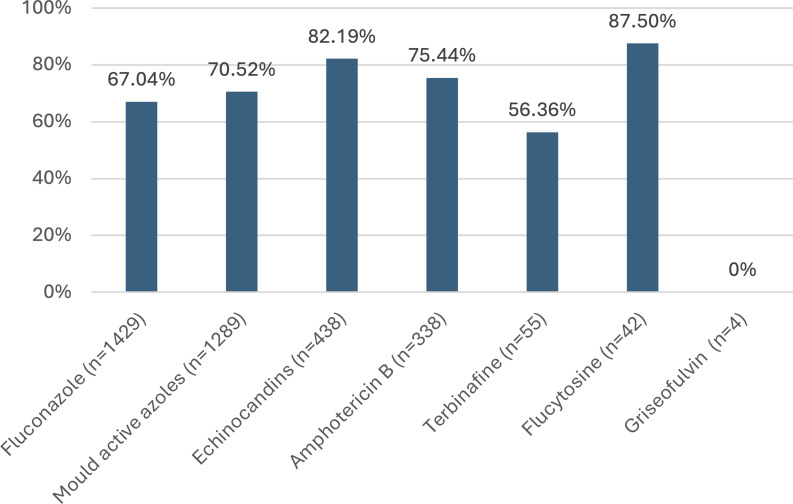
Rate of approval via local AMS process where required. Bar graph showing the percentage of antifungal prescriptions approved through the local Antimicrobial Stewardship (AMS) process, where approval was required.

Process measures included documentation of indication, documenation of a review or stop date, and approval for restricted antifungals according to local AMS processes. AMS approval rates varied by antifungal (Figure 5). The highest rates were observed for flucytosine and echinocandins, while the lowest were for terbinafine and fluconazole. Prescriptions that obtained approval had significantly higher appropriateness rates compared to those without approval (95.87% vs 82.93%, *P* < 0.001). This trend was consistent across all antifungals and classes assessed.

## Discussion

This study represents the first analysis of systemic antifungal prescribing quality using eleven years of national PPS data in Australia. To our knowledge, it constitutes the largest data set on antifungal prescribing quality published to date.

### Quality of prescribing and targets for improvement

This study found a high overall rate of appropriate prescribing for systemic antifungals, reflecting strong adherence to AMS principles across Australian hospitals. AMS programs are mandated under the Australian National Safety and Quality Health Service Standards ^17^ and form a core component of hospital accreditation. The high appropriateness rate observed suggests that these programs are effectively supporting high quality antifungal use. Notably, prescriptions with approval via local stewardship processes demonstrated higher appropriateness compared to those without approval, underscoring the value of stewardship interventions. This finding supports continued investment in AMS infrastructure.

Guideline compliance was also high, though generally lower than appropriateness. In hematology, bone marrow transplant, and solid organ transplant specialties, appropriateness and guideline compliance rates were closely aligned. This likely reflects the availability of detailed prescribing guidelines for these patient groups,^29–36^ the protocolized nature of prophylactic prescribing and the involvement of specialist clinicians in these high-risk populations.

Antifungal use was highest in principal referral hospitals, managing complex and immunocompromised patient populations. These hospitals also demonstrated the highest rates of appropriateness and guideline compliance, likely due to the presence of specialized teams and established protocols. In contrast, acute public hospitals, which provide a broad range of services but lack the same level of specialization, accounted for the second highest volume of antifungal prescribing but had the lowest rate of appropriateness. This may reflect more frequent prescribing in general medical and surgical units, where prescribing is less protocolized and national guidelines^26^ offer limited coverage.

Regional and remote hospitals demonstrated lower rates of appropriate prescribing, which may indicate disparities in access to infectious diseases expertise and AMS resources. These findings suggest a need for tailored support and capacity-building in non-metropolitan settings to ensure equitable stewardship practices nationwide.

Prescribing quality also varied by antifungal. Mold-active azoles, amphotericin B, and echinocandins—typically used in specialized settings—had high appropriateness, likely due to more stringent oversight and clearer guidelines. A national survey of Australian hospitals indicated that over 70% of surveyed facilities mandated preauthorization for these agents, with many conducting routine postprescription review and feedback (PPRF).^37^

Fluconazole, the most prescribed antifungal, had one of the lowest percentage appropriateness. This may reflect its broader use across diverse indications, some of which are less clearly defined or supported by guidelines. The same national survey ^37^ reported that fluconazole required preauthorization in fewer hospitals, and intravenous and oral fluconazole were excluded from expert PPRF in 26% and 42% of hospitals, respectively. Our study found that AMS approval for fluconazole was lower than for other agents, and prescriptions with approval had higher appropriateness. These findings suggest that fluconazole represents a key target for quality improvement, with enhanced stewardship oversight offering a potentially high-yield intervention.

Fluconazole use for urinary tract infections emerged as an area for improvement. Diagnostic stewardship strategies, such as including interpretive comments on microbiology reports may help educate clinicians and reduce inappropriate prescribing.^18^ Additionally, oral and dental indications often demonstrated prolonged therapy. Current guidelines recommend a duration of seven days for severe or refractory oropharyngeal candidiasis.^38^ Implementing PPRF for fluconazole prescriptions exceeding this duration may enhance appropriateness and reduce unnecessary exposure.

### Comparison to existing literature

The appropriateness rate observed in our study is notably higher than that reported in previous PPS studies evaluating antifungal prescribing across entire hospital populations. For instance, a multicenter study conducted in Greece ^8^ and a single-center study from India^9^ each reported overall appropriateness rates of approximately 75%. Similarly, a study conducted in a Spanish pediatric hospital found an appropriateness rate of 89%.^10^ In contrast, other observational studies assessing antifungal prescribing quality have focused on high-risk populations,^12,13,39^ such as hematology, oncology, or critical care settings, or on high-cost antifungals.^11,39^ These studies often excluded fluconazole and posaconazole from their assessments, and reported a broader range of appropriateness rates, from 38% to 92%. The higher appropriateness rate observed in our study may be attributable to differences in the definition of “appropriate therapy” and audit inclusion criteria. Additionally, the maturity of AMS programs in Australia may support more consistent and appropriate prescribing practices, potentially explaining the more favorable outcomes observed.

### Strengths of the Hospital NAPS tool for assessing antifungal prescribing quality

The Hospital NAPS is a well-established program that has demonstrated long-term sustainability at a national level, encompassing a wide range of health service types for over a decade. One of its key strengths lies in its dual approach to evaluating prescribing quality, encompassing both guideline compliance and appropriateness. In this study, we observed that appropriateness consistently exceeded guideline compliance. This finding highlights the added value of appropriateness assessments, which allow for a more nuanced evaluation of prescribing practices by accounting for patient-specific factors and clinical judgment, elements that guideline compliance alone may not capture. The Hospital NAPS appropriateness assessment matrix provides a robust and consistent framework for evaluating prescribing quality. It incorporates metrics found to be important and feasible in an international Delphi consensus survey on AFS metrics.^40^

### Considerations for the use of PPS methodology

The PPS methodology captures a snapshot of prescribing and antifungals represent a small proportion of the total data set. The methodology does not capture the dynamic progression of prescribing, making it difficult to assess the appropriateness across the full course of treatment. As a result, the sample size may be insufficient and the data set may not adequately reflect the complexities of antifungal prescribing, restricting its usefulness for developing targeted stewardship interventions.

In response to the limitations of the PPS methodology, the National Center for AMS and the National Center for Infections in Cancer have collaboratively developed and validated the Antifungal NAPS,^41^ a targeted, deep-dive audit integrated within the broader NAPS platform. This tool incorporates metrics deemed both important and feasible through international expert consensus, ^40^ enabling comprehensive assessment of antifungal prescribing across the entire course of therapy. It includes evaluation of infection risk factors and clinical outcomes. The Antifungal NAPS was piloted in 2023 in eleven Australian hospitals, revealing an appropriateness rate of 77.1%.^42^ This Figure is notably lower than the appropriateness observed in the current evaluation. We hypothesize that this discrepancy is in part attributable to the period prevalence methodology, which considers a broader range of criteria and evaluates each prescription in greater detail. Additionally, the number of hospitals participating in the Antifungal NAPS was far smaller than the Hospital NAPS so may not be representative of national prescribing.

### Limitations of this study

This study has several limitations that should be considered when interpreting the findings. First, temporal trends in the quality of antifungal prescribing were not assessed due to year-to-year variations in hospital participation which may confound the findings. Secondly, the evaluation of guideline compliance may be biased due to the high proportion of prescriptions classified as “directed therapy,” which were excluded from the guideline compliance analysis. Although these prescriptions were targeted toward identified organisms, relevant guidelines may still apply. This limitation has been acknowledged by the developers of the Hospital NAPS program and is a priority for future updates. Finally, topical antifungals were excluded. This decision was based on the focus of AFS interventions, which typically target systemic antifungals. Including topical prescriptions could obscure patterns relevant to systemic antifungal appropriateness.

## Conclusion

This study presents the first comprehensive national evaluation of systemic antifungal prescribing quality in Australia, drawing on over a decade of PPS data. The high rate of guideline compliance and appropriateness observed reflect the maturity and effectiveness of AMS programs. Nonetheless, notable variation in prescribing quality across hospital types, clinical specialties, and antifungals reveals important opportunities for targeted improvement. Fluconazole prescribing practices warrant enhanced stewardship oversight due to comparatively lower appropriateness.

The Hospital NAPS has provided a strong foundation for assessing antimicrobial prescribing practices at a national level. Building on this, the recently developed Antifungal NAPS offers a more tailored approach, enabling deeper insights into antifungal-specific prescribing and patient outcomes. This enhanced granularity may further support stewardship strategies and facilitate targeted interventions to optimize antifungal use in the hospital setting.

## Supporting information

10.1017/ash.2025.10214.sm001 Khanina et al. supplementary material Khanina et al. supplementary material

## Supplementary material

The supplementary material for this article can be found at https://doi.org/10.1017/ash.2025.10214.
