# Supplementary material for: Antifungal use and appropriateness: a study of Australian Hospital National Antimicrobial Prescribing Survey data
Source: Antimicrob Steward Healthc Epidemiol. 2025 Nov 17;5(1):e308. doi: 10.1017/ash.2025.10214 (PMC12645246; doi:10.1017/ash.2025.10214)
Supplement: Khanina et al. supplementary material [file S2732494X25102143sup001.pdf]

HOSPITAL NAPS National Antimicrobial Prescribing Survey

GUIDANCE | NCAS National Centre for Antimicrobial Stewardship

| Audit date | Patient identification number | Age / date of birth | Gender | Specialty ❒ *currently in ICU / NICU* | Ward | Weight *kg* | eGFR / CrCl *ml/min* |
|---|---|---|---|---|---|---|---|
| / / | | / / | M / F / O | | | | |

## Antimicrobials

*Only record the antimicrobials as prescribed at 8:00 am on the day of the audit and any surgical prophylaxis or stat doses in the previous 24 hours*

| For NICU patients Birth weight *kg* | Gestational age *weeks* |
|---|---|
| | |

| Start date | Antimicrobial | Route | Dose | Freq | Prescriber code# | Indication documented | Specify documented or presumed indication | Review / stop date documented | Guideline compliance (1-6) | Surgical prophylaxis > 24 hrs | Allergy mismatch | Microbiology mismatch | Indication does not require any antimicrobials | Incorrect route | Incorrect dose / frequency | Incorrect duration | Spectrum too broad | Spectrum too narrow | If restricted: approval given | Appropriateness (1- 5) |
|---|---|---|---|---|---|---|---|---|---|---|---|---|---|---|---|---|---|---|---|---|
| / / | | | | | | | | | | | | | | | | | | | | |
| / / | | | | | | | | | | | | | | | | | | | | |
| / / | | | | | | | | | | | | | | | | | | | | |
| / / | | | | | | | | | | | | | | | | | | | | |
| / / | | | | | | | | | | | | | | | | | | | | |

#Maximum of 6 characters, of which there must be at least 2 numbers

### Adverse drug reactions (including allergy) to antimicrobial

❒ Nil known ❒ Present ❒ Not documented

*If present, specify drugs or classes and nature of allergies*

### Were appropriate microbiology samples collected?

❒ Yes ❒ Partially* ❒ Not applicable ❒ No ❒ Not assessable

*Record the specimen type, organism, and susceptibilities if relevant*

*If more than one indication or microbiological sample is required

### Guideline compliance

1. Compliant with National Guidelines
2. Compliant with locally endorsed guidelines*
3. Non-compliant with guidelines
4. Directed therapy
5. No guidelines available
6. Not assessable

*Select **National Guidelines** if same as local guidelines

### Clinical notes or comments

❒ Renal replacement therapy given within previous 24hrs *(e.g., dialysis)*

### Surgical procedure if performed

If prophylaxis given within previous 24 hrs; *include in audit*

### Appropriateness

*please refer to the **appropriateness definitions** in the resources tab or in the user guide*

1. Optimal
2. Adequate
3. Suboptimal
4. Inadequate
5. Not assessable
